# Renal cell carcinoma with cavoatrial ventricular involvement

**DOI:** 10.1093/ehjcr/ytaf305

**Published:** 2025-06-27

**Authors:** Laurna McGovern, Roman Roy, Antonio Rullan

**Affiliations:** Cardiac Imaging Department, St Bartholomew’s Hospital, West Smithfield, London EC1A 7BE, UK; William Harvey Research Institute, National Institute for Health Research Barts Biomedical Research Centre, Queen Mary University of London, Charterhouse Square, London EC1M 6BQ, UK; Barts Heart Centre, St Bartholomew’s Hospital, Barts Health NHS Trust, West Smithfield, London EC1A 7BE, UK; Department of Oncology, The Royal Free NHS Foundation Trust, Pond Street, London NW3 2QG, UK; Institute of Immunity and Transplantation, University College London, The Pears Building, Pond Street, London NW3 2PP, UK

**Figure:**
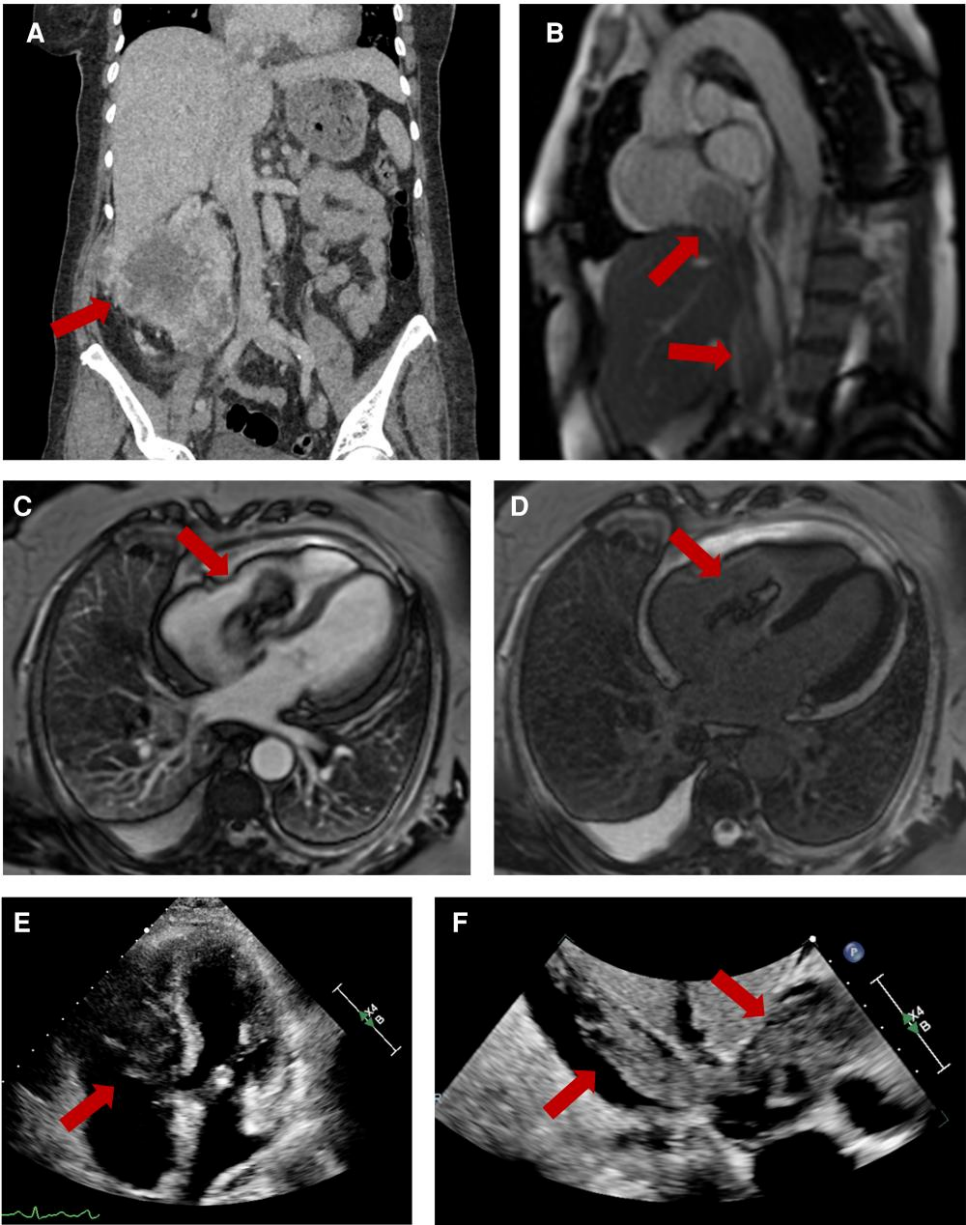


A 71-year-old woman with no significant past medical history presented to the emergency department with a 1-month history of worsening abdominal pain and bilateral lower-limb oedema. Computed tomography of chest, abdomen, and pelvis showed a large right renal mass (12 cm × 10 cm; *Panel A*), multiple lung metastasis, mediastinal, and right hilar adenopathy. Tumour thrombus was seen within the inferior vena cava (IVC) with extension to the right atrium and ventricle (*Panels E and F*). Cardiac magnetic resonance imaging (MRI) demonstrated a large, mobile mass (53 mm × 25 mm) extending along the whole IVC into the right atrium across the tricuspid valve into the right ventricle (*Panels B* and *C*; Supplementary material online, *Video S1*). There was no contrast uptake on first pass perfusion with heterogeneous late gadolinium enhancement in the periphery and central avascular core (*Panel D*). The patient was asymptomatic at diagnosis, with mild–moderate tricuspid regurgitation and preserved biventricular systolic function. She was started on anticoagulation with heparin.

Right renal biopsy confirmed Grade 4 clear cell renal cell carcinoma (RCC; AE1/AE3, CD10 and PAX-8 positive) with areas of necrosis and sarcomatoid differentiation. Following cardio-oncology multidisciplinary team discussion, treatment with ipilimumab and nivolumab was commenced. Unfortunately, the patient experienced disease progression to first-line treatment, developed significant right-sided cardiac failure, and died 3 months after diagnosis.

Intravascular invasion of the IVC complicates 5%–15% of RCC cases with 1% involving extension to the right atrium. Right ventricular involvement is even less frequent, with case reports of right ventricular outflow tract obstruction published in the literature. The median overall survival for patients with metastatic RCC after first-line treatment is 4 years. Although echocardiography is commonly the first line in assessing cardiac masses, cardiac MRI is particularly valuable in characterizing their aetiology.

(*A*) Computed tomography: coronal view of the primary renal cell carcinoma. (*B*) Cardiac magnetic resonance: Sagittal view of tumour/thrombus in inferior vena cava extending to right atrium. (*C*) Cardiac magnetic resonance: four-chamber early gadolinium enhanced image showing a large, mobile mass (53 mm × 25 mm) in the right atrium extending across the tricuspid valve into the right ventricle. (*D*) Cardiac magnetic resonance: four-chamber late gadolinium image demonstrating heterogeneous late gadolinium enhancement in the periphery and central avascular core. (*E*) Echocardiogram: apical four-chamber view of tumour thrombus in right ventricle. (*F*) Echocardiogram: subcostal view of tumour thrombus extending from inferior vena cava to right atrium.

## Supplementary Material

ytaf305_Supplementary_Data

## Data Availability

The data underlying this article are available in the article and in its online Supplementary material.

